# 3,3′-Bis(4-nitro­phen­yl)-1,1′-(*p*-phenyl­ene)dithio­urea dimethyl sulfoxide disolvate

**DOI:** 10.1107/S160053680801430X

**Published:** 2008-05-17

**Authors:** Wen-Kui Dong, Hai-Bo Yan, Lan-Qin Chai, Zhong-Wu Lv, Chun-Yu Zhao

**Affiliations:** a School of Chemical and Biological Engineering, Lanzhou Jiaotong University, Lanzhou 730070, People’s Republic of China

## Abstract

The asymmetric unit of the title compound, C_22_H_16_N_6_O_6_S_2_·2C_2_H_6_OS, consists of one half-mol­ecule of the centrosymmetric thiourea derivative and one molecule of dimethyl sulfoxide (DMSO). The carbonyl group forms an intra­molecular hydrogen bond with the NH group, creating a six-membered (C—N—C—N—H⋯O) ring. Two other N—H⋯O hydro­gen bonds link one mol­ecule of the thio­urea to two mol­ecules of DMSO.

## Related literature

For related literature, see: Burrows *et al.* (1997 ▶); Dong *et al.* (2006 ▶, 2007 ▶); Foss *et al.* (2004 ▶); Valdés-Martínez *et al*. (2000 ▶, 2004 ▶); Zhang *et al.* (2006 ▶); Huang *et al.* (2006 ▶).
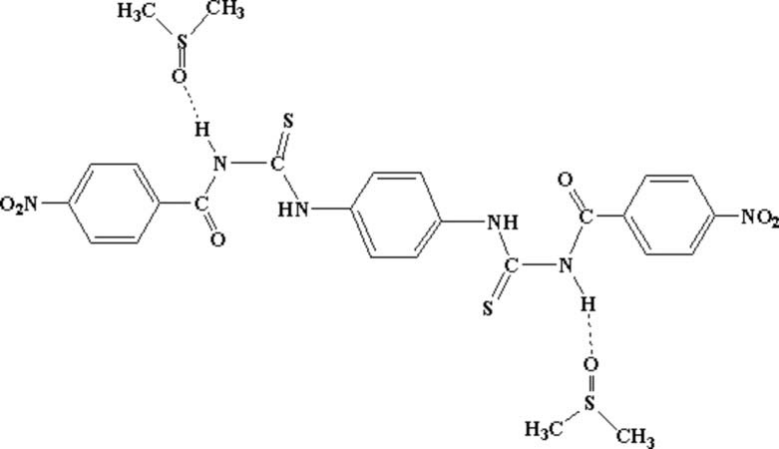


## Experimental

### 

#### Crystal data

C_22_H_16_N_6_O_6_S_2_·2C_2_H_6_OS
*M* *_r_* = 680.78
Monoclinic, 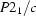

*a* = 11.6949 (18) Å
*b* = 6.6916 (11) Å
*c* = 20.449 (2) Å
β = 106.353 (2)°
*V* = 1535.5 (4) Å^3^
*Z* = 2
Mo *K*α radiation
μ = 0.37 mm^−1^
*T* = 298 (2) K
0.33 × 0.17 × 0.11 mm

#### Data collection

Bruker SMART CCD area-detector diffractometer
Absorption correction: multi-scan (*SADABS*; Sheldrick, 1996 ▶) *T*_min_ = 0.888, *T*_max_ = 0.961
7318 measured reflections
2684 independent reflections
1547 reflections with *I* > 2σ(*I*)
*R*_int_ = 0.097

#### Refinement

*R*[*F* ^2^ > 2σ(*F* ^2^)] = 0.069
*wR*(*F* ^2^) = 0.199
*S* = 0.96
2684 reflections
199 parameters
H-atom parameters constrained
Δρ_max_ = 0.44 e Å^−3^
Δρ_min_ = −0.43 e Å^−3^

### 

Data collection: *SMART* (Siemens, 1996 ▶); cell refinement: *SAINT* (Siemens, 1996 ▶); data reduction: *SAINT*; program(s) used to solve structure: *SHELXS97* (Sheldrick, 2008 ▶); program(s) used to refine structure: *SHELXL97* (Sheldrick, 2008 ▶); molecular graphics: *SHELXTL* (Sheldrick, 2008 ▶); software used to prepare material for publication: *SHELXTL*.

## Supplementary Material

Crystal structure: contains datablocks global, I. DOI: 10.1107/S160053680801430X/fl2195sup1.cif

Structure factors: contains datablocks I. DOI: 10.1107/S160053680801430X/fl2195Isup2.hkl

Additional supplementary materials:  crystallographic information; 3D view; checkCIF report

## Figures and Tables

**Table 1:** Hydrogen-bond geometry (Å, °)

*D*—H⋯*A*	*D*—H	H⋯*A*	*D*⋯*A*	*D*—H⋯*A*
N1—H1⋯O4	0.86	2.09	2.942 (5)	169
N2—H2⋯O1	0.86	1.84	2.579 (5)	143

**Fig. 1.**
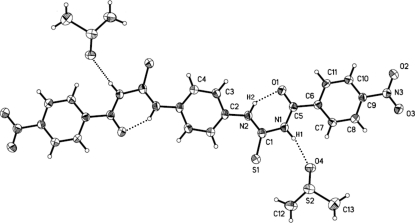
Molecule structure of (I) with the atom numbering. Displacement ellipsoids for non-H atoms are drawn at the 30% probability level.

## supplementary crystallographic information

### Comment

Thiourea and its derivatives are of interest to us because of their varied biological activity as well as their ability to form strong H bonds as both donors and acceptors (Valdés-Martínez, *et al.*, 2000; Jesus, *et al.*, 2004, Burrows *et al.*, 1997), and their tendency to coordinate with metal ions (Huang, *et al.*, 2006; Foss, *et al.*, 2004). In recent years, thioureas have been recognized as important neutral receptors because of their anion recognition properties (Zhang, *et al.*, 2006) and for their ability to easily form intramolecular hydrogen bonds such as between the benzoyl (CO) and the N—H group of acylthioureas (Dong *et al.*, 2006). In continuation of our previous studies on the synthesis and structural characterization of *N*-benzoyl-*N*'-(3-pyridyl)thiourea (II) (Dong, *et al.*, 2006) and *N*,*N*'-(1,6-hexamethylene)-bis(benzoylthiourea) (Dong, *et al.*, 2007), a novel bisbenzoylthiourea which crystallized as a dimethyl sulfoxide disolvate (I) has now been synthesized and structurally characterized.

The crystal structure of (I) is built up by one N, *N*'-(*p*-phenyl)-bis(*p*-nitro)benzoylthiourea molecule and two dimethyl sulfoxide solvent molecules. The carbonyl group of the thiourea forms an intramolecular hydrogen bond with the N—H group to form a six-membered (C/N/C/N/H/O) ring. The C=O bond length at 1.225 (5)Å is longer than the average C=O bond length (1.200 Å). This is most likely due to the intramolecular hydrogen bonding which is similar to the situation found in the structure of (II) (Dong, *et al.*, 2006).

There are also N—H···O hydrogen bonds between N1 of the thiourea and the O atoms of the DMSO S=O groups which, together with other intermolecular C—H···O and C—H···S interactions, stabilize the three-dimensional structure of (I).

### Experimental

(*p*-Nitro)benzoyl chloride (1.86 g, 10 mmol) was reacted with ammonium thiocyanate (1.14 g, 15 mmol) in CH_2_Cl_2_ (25 ml) solution under solid-liquid phase transfer catalysis, using polyethylene glycol-400 (0.18 g) as the catalyst, to give the corresponding (*p*-nitro)benzoyl isothiocyanate under stirring at the room temperature. This was followed by slow addition of 15 ml CH_2_Cl_2_ solution dissolved *p*-phenylenediamine (1.60 g, 0.01 mmol). The corresponding yellow compound precipitated immediately. The product was filtered, washed with water and CH_2_Cl_2_, dried, and recrystallized from THF to give the titled thiourea. Yield, 72.6%. m. p. 243 - 244 °C. Anal. Calc. for C_22_H_16_N_6_O_6_S_2_ (%): C, 50.38; H, 3.07; N, 16.02. Found: C, 50.27; H, 3.15; N, 15.99. Selected IR data (cm^-1 ^, KBr pellet): 3341, 3191 (ν NH), 1675 (ν C=O), 1152 (ν C=S). ^1^H NMR (400 MHz, DMSO-d6, δ, p.p.m.): 8.05 (d, J = 17.2 Hz, 4H, ArH), 8.19 (d, J = 8.2 Hz, 4H, ArH), 8.34 (dd, J = 17.2, 7.2 Hz, 4H, ArH), 11.98 (s, 2H, NH), 12.42 (s, 2H, NH).

A DMSO solution of the thiourea was placed in a hexane atmosphere, after about one week, along with diffusion of hexane into the DMSO solution, yellow needle-shaped single crystals suitable for X-ray crystallographic analysis were obtained.

### Refinement

Non-H atoms were refined anisotropically. H atoms were treated as riding atoms with distances C—H = 0.96(CH_2_), or 0.93Å (CH),*O*—H = 0.86 Å, and *U*_iso_(H) = 1.2*U*_eq_(C) and 1.5*U*_eq_(O).

### Crystal data

**Table:**

C_22_H_16_N_6_O_6_S_2_·2C_2_H_6_OS	*F*_000_ = 708
*M**_r_* = 680.78	*D*_x_ = 1.472 Mg m^−^^3^
Monoclinic, *P*2_1_/*c*	Mo *K*α radiation λ = 0.71073 Å
*a* = 11.6949 (18) Å	Cell parameters from 1517 reflections
*b* = 6.6916 (11) Å	θ = 3.3–25.3º
*c* = 20.449 (2) Å	µ = 0.37 mm^−^^1^
β = 106.353 (2)º	*T* = 298 (2) K
*V* = 1535.5 (4) Å^3^	Needle-shaped, yellow
*Z* = 2	0.33 × 0.17 × 0.11 mm

### Data collection

**Table:**

Bruker SMART CCD area-detector diffractometer	2684 independent reflections
Radiation source: fine-focus sealed tube	1547 reflections with *I* > 2σ(*I*)
Monochromator: graphite	*R*_int_ = 0.097
*T* = 298(2) K	θ_max_ = 25.0º
φ and ω scans	θ_min_ = 1.8º
Absorption correction: multi-scan(SADABS; Sheldrick, 1996)	*h* = −11→13
*T*_min_ = 0.888, *T*_max_ = 0.961	*k* = −7→7
7318 measured reflections	*l* = −24→24

### Refinement

**Table:**

Refinement on *F*^2^	Secondary atom site location: difference Fourier map
Least-squares matrix: full	Hydrogen site location: inferred from neighbouring sites
*R*[*F*^2^ > 2σ(*F*^2^)] = 0.069	H-atom parameters constrained
*wR*(*F*^2^) = 0.199	*w* = 1/[σ^2^(*F*_o_^2^) + (0.1071*P*)^2^] where *P* = (*F*_o_^2^ + 2*F*_c_^2^)/3
*S* = 0.97	(Δ/σ)_max_ < 0.001
2684 reflections	Δρ_max_ = 0.44 e Å^−^^3^
199 parameters	Δρ_min_ = −0.43 e Å^−^^3^
Primary atom site location: structure-invariant direct methods	Extinction correction: none

### Special details

**Table:**

Geometry. All e.s.d.'s (except the e.s.d. in the dihedral angle between two l.s. planes) are estimated using the full covariance matrix. The cell e.s.d.'s are taken into account individually in the estimation of e.s.d.'s in distances, angles and torsion angles; correlations between e.s.d.'s in cell parameters are only used when they are defined by crystal symmetry. An approximate (isotropic) treatment of cell e.s.d.'s is used for estimating e.s.d.'s involving l.s. planes.
Refinement. Refinement of *F*^2^ against ALL reflections. The weighted *R*-factor *wR* and goodness of fit *S* are based on *F*^2^, conventional *R*-factors *R* are based on *F*, with *F* set to zero for negative *F*^2^. The threshold expression of *F*^2^ > σ(*F*^2^) is used only for calculating *R*-factors(gt) *etc*. and is not relevant to the choice of reflections for refinement. *R*-factors based on *F*^2^ are statistically about twice as large as those based on *F*, and *R*- factors based on ALL data will be even larger.

### Fractional atomic coordinates and isotropic or equivalent isotropic displacement parameters (Å^2^)

**Table:**

	*x*	*y*	*z*	*U*_iso_*/*U*_eq_	
N1	0.7828 (3)	0.3522 (5)	0.51603 (16)	0.0505 (8)	
H1	0.7673	0.2724	0.5454	0.061*	
N2	0.8719 (3)	0.6436 (5)	0.49516 (16)	0.0507 (9)	
H2	0.8343	0.6109	0.4541	0.061*	
N3	0.4791 (3)	−0.4240 (5)	0.36972 (18)	0.0565 (9)	
O1	0.7411 (3)	0.4141 (5)	0.40259 (14)	0.0614 (8)	
O2	0.4552 (3)	−0.4678 (5)	0.31005 (16)	0.0733 (10)	
O3	0.4566 (3)	−0.5286 (5)	0.41241 (17)	0.0776 (10)	
O4	0.7382 (3)	0.1230 (6)	0.62917 (14)	0.0782 (10)	
S1	0.91836 (14)	0.5258 (2)	0.62543 (6)	0.0858 (6)	
S2	0.82807 (12)	0.0691 (2)	0.69426 (6)	0.0711 (5)	
C1	0.8572 (4)	0.5173 (6)	0.5429 (2)	0.0502 (10)	
C2	0.9391 (3)	0.8218 (6)	0.5010 (2)	0.0454 (10)	
C3	0.9492 (4)	0.9013 (7)	0.4410 (2)	0.0557 (11)	
H3	0.9141	0.8344	0.4004	0.067*	
C4	1.0090 (4)	1.0755 (7)	0.4389 (2)	0.0562 (11)	
H4	1.0150	1.1251	0.3975	0.067*	
C5	0.7326 (3)	0.3047 (6)	0.44901 (19)	0.0455 (10)	
C6	0.6642 (3)	0.1137 (6)	0.43250 (18)	0.0443 (9)	
C7	0.6443 (4)	−0.0164 (6)	0.4804 (2)	0.0501 (10)	
H7	0.6723	0.0141	0.5265	0.060*	
C8	0.5824 (4)	−0.1933 (7)	0.4596 (2)	0.0548 (11)	
H8	0.5674	−0.2810	0.4915	0.066*	
C9	0.5441 (3)	−0.2359 (6)	0.39199 (19)	0.0461 (10)	
C10	0.5631 (4)	−0.1108 (7)	0.3439 (2)	0.0600 (12)	
H10	0.5363	−0.1438	0.2979	0.072*	
C11	0.6225 (4)	0.0652 (7)	0.3643 (2)	0.0592 (12)	
H11	0.6349	0.1531	0.3318	0.071*	
C12	0.8181 (5)	0.2507 (9)	0.7540 (2)	0.0870 (17)	
H12A	0.8448	0.3769	0.7414	0.131*	
H12B	0.8671	0.2124	0.7983	0.131*	
H12C	0.7368	0.2624	0.7550	0.131*	
C13	0.7650 (6)	−0.1309 (11)	0.7298 (3)	0.120 (2)	
H13A	0.6928	−0.0866	0.7388	0.180*	
H13B	0.8206	−0.1736	0.7716	0.180*	
H13C	0.7477	−0.2405	0.6983	0.180*	

### Atomic displacement parameters (Å^2^)

**Table:**

	*U*^11^	*U*^22^	*U*^33^	*U*^12^	*U*^13^	*U*^23^
N1	0.050 (2)	0.046 (2)	0.0538 (19)	−0.0108 (17)	0.0109 (15)	0.0002 (17)
N2	0.0460 (19)	0.047 (2)	0.0551 (19)	−0.0107 (16)	0.0077 (15)	−0.0008 (17)
N3	0.064 (2)	0.048 (2)	0.055 (2)	−0.0041 (18)	0.0123 (18)	0.0011 (18)
O1	0.0683 (19)	0.057 (2)	0.0569 (17)	−0.0188 (15)	0.0140 (14)	0.0064 (15)
O2	0.094 (2)	0.065 (2)	0.0564 (19)	−0.0207 (18)	0.0137 (17)	−0.0127 (16)
O3	0.100 (3)	0.064 (2)	0.064 (2)	−0.0294 (19)	0.0153 (18)	0.0067 (17)
O4	0.090 (2)	0.090 (3)	0.0495 (18)	−0.021 (2)	0.0117 (16)	0.0026 (17)
S1	0.1133 (12)	0.0803 (10)	0.0544 (8)	−0.0440 (9)	0.0080 (7)	−0.0012 (6)
S2	0.0732 (9)	0.0804 (10)	0.0590 (7)	0.0026 (7)	0.0174 (6)	−0.0010 (6)
C1	0.043 (2)	0.048 (3)	0.059 (3)	−0.0024 (19)	0.0118 (19)	−0.002 (2)
C2	0.035 (2)	0.040 (2)	0.060 (2)	−0.0015 (17)	0.0101 (17)	0.0030 (19)
C3	0.059 (3)	0.049 (3)	0.054 (2)	−0.009 (2)	0.0082 (19)	−0.007 (2)
C4	0.064 (3)	0.055 (3)	0.050 (2)	−0.011 (2)	0.016 (2)	−0.002 (2)
C5	0.035 (2)	0.048 (3)	0.050 (2)	−0.0030 (18)	0.0056 (17)	0.001 (2)
C6	0.040 (2)	0.047 (2)	0.046 (2)	−0.0016 (18)	0.0121 (17)	0.0012 (18)
C7	0.051 (2)	0.053 (3)	0.042 (2)	−0.006 (2)	0.0060 (18)	−0.0026 (19)
C8	0.062 (3)	0.052 (3)	0.053 (2)	−0.008 (2)	0.021 (2)	0.004 (2)
C9	0.046 (2)	0.042 (2)	0.047 (2)	−0.0039 (18)	0.0067 (17)	0.0017 (19)
C10	0.073 (3)	0.061 (3)	0.042 (2)	−0.018 (2)	0.011 (2)	−0.001 (2)
C11	0.073 (3)	0.060 (3)	0.046 (2)	−0.023 (2)	0.017 (2)	0.008 (2)
C12	0.101 (4)	0.097 (4)	0.059 (3)	0.013 (3)	0.016 (3)	−0.001 (3)
C13	0.158 (6)	0.091 (5)	0.100 (4)	−0.022 (5)	0.020 (4)	0.024 (4)

### Geometric parameters (Å, °)

**Table:**

N1—C5	1.368 (4)	C4—H4	0.9300
N1—C1	1.418 (5)	C5—C6	1.495 (6)
N1—H1	0.8600	C6—C7	1.379 (6)
N2—C1	1.338 (5)	C6—C11	1.380 (5)
N2—C2	1.414 (5)	C7—C8	1.390 (6)
N2—H2	0.8600	C7—H7	0.9300
N3—O3	1.204 (4)	C8—C9	1.358 (5)
N3—O2	1.209 (4)	C8—H8	0.9300
N3—C9	1.475 (5)	C9—C10	1.356 (6)
O1—C5	1.225 (5)	C10—C11	1.371 (6)
O4—S2	1.490 (3)	C10—H10	0.9300
S1—C1	1.638 (4)	C11—H11	0.9300
S2—C12	1.750 (5)	C12—H12A	0.9600
S2—C13	1.779 (6)	C12—H12B	0.9600
C2—C3	1.372 (5)	C12—H12C	0.9600
C2—C4^i^	1.389 (5)	C13—H13A	0.9600
C3—C4	1.367 (6)	C13—H13B	0.9600
C3—H3	0.9300	C13—H13C	0.9600
C4—C2^i^	1.389 (5)		
			
C5—N1—C1	127.8 (3)	C11—C6—C5	116.4 (3)
C5—N1—H1	116.1	C6—C7—C8	119.9 (4)
C1—N1—H1	116.1	C6—C7—H7	120.1
C1—N2—C2	130.8 (3)	C8—C7—H7	120.1
C1—N2—H2	114.6	C9—C8—C7	119.0 (4)
C2—N2—H2	114.6	C9—C8—H8	120.5
O3—N3—O2	123.7 (4)	C7—C8—H8	120.5
O3—N3—C9	118.1 (3)	C10—C9—C8	122.3 (4)
O2—N3—C9	118.1 (4)	C10—C9—N3	118.6 (3)
O4—S2—C12	106.6 (2)	C8—C9—N3	119.2 (4)
O4—S2—C13	106.1 (2)	C9—C10—C11	118.8 (4)
C12—S2—C13	96.9 (3)	C9—C10—H10	120.6
N2—C1—N1	113.6 (3)	C11—C10—H10	120.6
N2—C1—S1	128.4 (3)	C10—C11—C6	121.0 (4)
N1—C1—S1	117.9 (3)	C10—C11—H11	119.5
C3—C2—C4^i^	118.2 (4)	C6—C11—H11	119.5
C3—C2—N2	115.9 (4)	S2—C12—H12A	109.5
C4^i^—C2—N2	125.8 (4)	S2—C12—H12B	109.5
C4—C3—C2	122.1 (4)	H12A—C12—H12B	109.5
C4—C3—H3	118.9	S2—C12—H12C	109.5
C2—C3—H3	118.9	H12A—C12—H12C	109.5
C3—C4—C2^i^	119.6 (4)	H12B—C12—H12C	109.5
C3—C4—H4	120.2	S2—C13—H13A	109.5
C2^i^—C4—H4	120.2	S2—C13—H13B	109.5
O1—C5—N1	122.2 (4)	H13A—C13—H13B	109.5
O1—C5—C6	119.4 (3)	S2—C13—H13C	109.5
N1—C5—C6	118.4 (4)	H13A—C13—H13C	109.5
C7—C6—C11	119.1 (4)	H13B—C13—H13C	109.5
C7—C6—C5	124.5 (3)		
			
C2—N2—C1—N1	−179.4 (4)	C11—C6—C7—C8	−0.3 (6)
C2—N2—C1—S1	−2.6 (7)	C5—C6—C7—C8	−178.1 (4)
C5—N1—C1—N2	4.2 (6)	C6—C7—C8—C9	1.0 (6)
C5—N1—C1—S1	−173.0 (3)	C7—C8—C9—C10	−0.7 (7)
C1—N2—C2—C3	170.9 (4)	C7—C8—C9—N3	179.6 (4)
C1—N2—C2—C4^i^	−11.5 (7)	O3—N3—C9—C10	−175.8 (4)
C4^i^—C2—C3—C4	0.6 (7)	O2—N3—C9—C10	6.8 (6)
N2—C2—C3—C4	178.4 (4)	O3—N3—C9—C8	4.0 (6)
C2—C3—C4—C2^i^	−0.6 (7)	O2—N3—C9—C8	−173.4 (4)
C1—N1—C5—O1	−5.3 (6)	C8—C9—C10—C11	−0.3 (7)
C1—N1—C5—C6	175.5 (4)	N3—C9—C10—C11	179.4 (4)
O1—C5—C6—C7	−177.0 (4)	C9—C10—C11—C6	1.1 (7)
N1—C5—C6—C7	2.3 (6)	C7—C6—C11—C10	−0.7 (7)
O1—C5—C6—C11	5.2 (6)	C5—C6—C11—C10	177.2 (4)
N1—C5—C6—C11	−175.6 (4)		

Symmetry codes: (i) −*x*+2, −*y*+2, −*z*+1.

### Hydrogen-bond geometry (Å, °)

**Table:**

*D*—H···*A*	*D*—H	H···*A*	*D*···*A*	*D*—H···*A*
N1—H1···O4	0.86	2.09	2.942 (5)	169
N2—H2···O1	0.86	1.84	2.579 (5)	143
